# Patterns of Gender Equality at Workplaces and Psychological Distress

**DOI:** 10.1371/journal.pone.0053246

**Published:** 2013-01-09

**Authors:** Sofia Elwér, Lisa Harryson, Malin Bolin, Anne Hammarström

**Affiliations:** 1 Department of Public Health and Clinical Medicine, Family Medicine, Umeå University, Umeå, Sweden; 2 National Graduate School for Gender Studies at Umeå Centre for Gender Studies, Umeå University, Umeå, Sweden; 3 Department of Social Sciences, Mid Sweden University, Sundsvall, Sweden; Vanderbilt University, United States of America

## Abstract

Research in the field of occupational health often uses a risk factor approach which has been criticized by feminist researchers for not considering the combination of many different variables that are at play simultaneously. To overcome this shortcoming this study aims to identify patterns of gender equality at workplaces and to investigate how these patterns are associated with psychological distress. Questionnaire data from the Northern Swedish Cohort (n = 715) have been analysed and supplemented with register data about the participants' workplaces. The register data were used to create gender equality indicators of women/men ratios of number of employees, educational level, salary and parental leave. Cluster analysis was used to identify patterns of gender equality at the workplaces. Differences in psychological distress between the clusters were analysed by chi-square test and logistic regression analyses, adjusting for individual socio-demographics and previous psychological distress. The cluster analysis resulted in six distinctive clusters with different patterns of gender equality at the workplaces that were associated to psychological distress for women but not for men. For women the highest odds of psychological distress was found on traditionally gender unequal workplaces. The lowest overall occurrence of psychological distress as well as same occurrence for women and men was found on the most gender equal workplaces. The results from this study support the convergence hypothesis as gender equality at the workplace does not only relate to better mental health for women, but also more similar occurrence of mental ill-health between women and men. This study highlights the importance of utilizing a multidimensional view of gender equality to understand its association to health outcomes. Health policies need to consider gender equality at the workplace level as a social determinant of health that is of importance for reducing differences in health outcomes for women and men.

## Introduction

All over the world the labour market is strongly gender segregated, leaving women and men exposed to different work environments in different occupations (horizontal segregation) and hierarchical positions (vertical segregation), with consequences for women's and men's health status [1]. However, the gender segregated labour market is not the only aspect of gender inequalities with an impact on health status. In fact, gender equality is multidimensional and includes several dimensions of gender relations in division of labour, emotions, symbolic representations as well as power and decision making [2]. Within an organization these patterns of gender relations constitutes a gender regime and can include inequalities between women and men in the shape of discrimination in relation to opportunities, access to services and allocation of resources or benefits [3]. All of these aspects of gender inequalities influence women's and men's working life and can lead to gendered experiences of somatic and mental health status [1]. Gender inequalities can therefore be seen as social obstacles that prevent fairness in health status between women and men making it an important issue for public health research [4]. Previous research in the field of occupational health has often used a risk factor approach to health outcomes which has been criticized by feminist researchers for not considering the combination of many different variables that are at play simultaneously [5]. A contextual approach that includes many different aspects of gender equality can therefore add new perspectives that enable us to grasp how gender equality at the workplace relate to health status. We understand gender as a social relational process that is being constructed in women's and men's everyday life [6], [7].When approaching the area of gender equality, work and health experiences, an underlying premise of our research is the notion that similar life circumstances for women and men would lead to similar health outcomes, sometimes referred to as the convergence theory [8]–[11]. This paper will explore three hypotheses:

- Patterns of gender equality at the workplace are related to mental health for both women and men.
- Similar work conditions for women and men is related to a convergence of health outcomes.
- Gender equality is multidimensional and therefore the combination of several aspects of gender equality needs to be taken into account to understand its relation to health outcomes.

To introduce the field we will now describe some of the aspects of gender equality that have been analysed in relation to health status in previous research: education, salary, parental leave and proportion of women and men at the workplace [12]–[14].

### Aspects of gender equality in relation to health status

An equal *proportion of women and men* at the workplace indicates that the general requirements for the work acknowledge both women's and men's potential. In the Swedish labour market only about 10 percent of the workers are in an occupation where women and men are equally represented [15]. Research on gender segregated workplaces often refers to workplaces as *women dominated* when women are in the majority. However, as the word dominate is related to power, and it is not certain that power is connected to being in the majority, we use the term majority. Studies from Sweden and the UK have shown a higher risk of physical and mental illness among women working in occupations with a majority of men [14], [16], and better health status is reported in those few occupations that are gender-integrated [17]. However, a limitation in previous research is that a majority of the studies of horizontal gender segregation and health status have concerned occupations, rather than workplaces [14], [17], which means that the specific work environmental influence on health status is not considered [18]. One of the few studies with a workplace focus found that the proportion of women and men was only related to poor self-rated health for men, with more ill-health among men working at workplaces with a majority of women [17].

An equal *educational level* and *salary* between women and men at a workplace suggests that women and men have similar status and power. In Sweden, more women than men have a post-secondary education but still women earn only 84 percent of what men earn. After adjustment for age, education, working time, sector and profession, women earn 92 percent of what men earn [15]. High educational level and income have been shown to be associated with lower risk of morbidity and mortality in Western societies [19], [20], which can be explained by better work and economic conditions, psychosocial resources and healthy lifestyles [21]. However, economic and work-related benefits of education and income can have different meanings due to gender and cultural context [12].

Gendered patterns of *parental leave*, in terms of the relation between women's and men's use of parental leave at the workplace, can give an indication of gendered differences in family responsibility. In Sweden parents are entitled to 480 days of parental leave for each child. Sixty days are reserved for each parent and the rest of the days can be used by either parent until the child is 8 years old. For children under 12 years of age both parents also have the right to take temporary parental leave to care for sick children. Women use 78 percent of the parental leave days and 65 percent of the temporary parental leave [15]. The workplace has been acknowledged as an important actor for understanding the gendered division of childcare and parental leave and men's lower levels of parental leave in Sweden have partly been explained by workplace factors such as organizational culture [22]–[24]. However, the relationship between gender equality in parental leave and health status is mainly studied with regard to the division between parents. Fathers taking parental leave more than 30 days have a decreased risk of all-cause mortality [13], whereas mothers taking fewer days of parental leave than their partner have a higher risk of death and sickness compared to other mothers [10]. Furthermore, Swedish fathers sharing parental leave equally with their partner and living in an equal municipality have lower levels of sick leave, while those who are less equal than their municipality fare worse [25]. In the same study results for women show that mothers taking the larger part of parental leave and also living in a gender unequal municipality have the lowest levels of sick leave whereas pioneers have the highest levels.

### Patterns of gender equality

In previous research aspects of gender equality in relation to health status discussed above has not been analysed as a part of a workplace gender regime. Few attempts have been made to analyse how several dimensions of gender equality at workplaces are related to employee's health status. One such attempt is a Swedish study in which an index has been constructed that summarizes several dimensions of gender gaps in organizations showing that companies with small gender gaps have a more gender-equal distribution of sickness absence [26]. To further understand what constitutes gender-equal workplaces and their relation to health status it is important to also analyse how the different aspects of gender equality shape patterns and to acknowledge that gender inequality can take two directions: discriminating against women or against men.

Thus, previous research on gender equality and health status has not focused on the workplace level. For example, studies about the importance of work-place patterns of gendered use of parental leave for individual health status is missing from the literature. There is also a lack of research related to health including several dimensions of gender equality at the workplace that are at play simultaneously. Another limitation of previous research is that possible health selection has not been taken into account because of a lack of longitudinal studies [17], [27]. To fill these research gaps we have conducted a contextual analysis exploring how the overall gender equality patterns of workplaces are related to psychological distress among women and men, taking health-related selection into account. The setting of the present study is Sweden which is adequate for several reasons. First, women and men in Sweden participate in the paid workforce to almost the same extent. Second, in Sweden there is a broad public and political support for promoting gender equality in working life with a discriminations act that requires each employer with more than 25 employees to have a gender equality plan that prevent sex discrimination and promote gender equality [28]. Third, Sweden has a progressive parental leave legislation that enables parental leave for both mothers and fathers. And fourth, Sweden also has a thorough register data on work place factors on an individual level.

The aim of this study was twofold:

1. To identify patterns of gender equality at workplaces.
2. To investigate how patterns of gender equality at the workplaces are associated with psychological distress among women and men.

## Methods

### Ethics statement

The Regional Ethical Review Board in Umeå, Sweden, has approved this study. According to Swedish law, written consent is not requested in this type of studies. The participants are regarded as giving consent when they send in their questionnaire or are willing to participate in interviews. The responders are always clearly informed that the participation is voluntary and that they can withdraw from the study whenever they wish at their discretion.

### Population and data collection

The data collection for this study was performed in two steps. First, data were collected for the Northern Swedish Cohort which consists of all pupils (506 girls and 577 boys) who studied in their last year of compulsory school in a medium-sized Swedish industrial town in 1981 [29]. Regardless of where the participants had moved after 1981 they were followed with comprehensive questionnaire investigations concerning school, employment, socioeconomic conditions, health status and health behaviour. The cohort has been followed up in 1983 (age 18), 1986 (age 21), 1995 (age 30) and 2007 (age 42). In this study, data from the last follow up in 2007 when the participants 42 years old has been analysed. In order to adjust for previous mental health status, data from when the participants were 21 years old before becoming established in the labour market, has also been used. The response rate (in relation to those still alive in the original cohort) was 98% in 1986 and 94% in 2007 (n = 1010).

**Figure 1:**
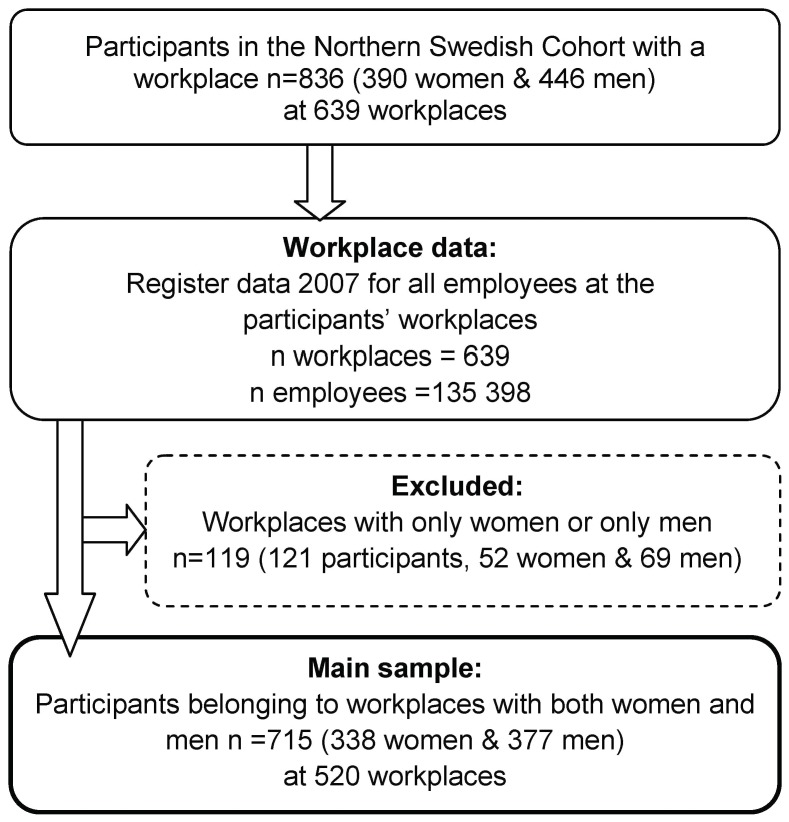
Sample procedure.

In a second step of the data collection the questionnaire data from the Northern Swedish Cohort were supplemented with register data for all participants in the cohort belonging to a workplace in Sweden at age 42 (n = 837). For each participant's workplace, register data about gender, salary, education, parental leave, temporary parental leave and age for all employees (n = 135 398) was collected from Statistics Sweden's Longitudinal Integration Database for Health Insurance and Labour Market Studies (LISA). As the aim of this study was to analyse patterns of gender inequality, workplaces with only men or women were excluded. This resulted in a dataset with a main sample of 715 participants from the Northern Swedish Cohort belonging to 520 workplaces, complemented with aggregated register data for the workplaces based on data for 134 450 employees. The sample procedure is presented in Figure 1.

### Indicators of gender equality

Five indicators of gender equality were created through aggregating workplace data and calculating the women/men ratio for: (I) number of employees; (II) mean net salary; (III) mean educational level; (IV) mean net days of parental leave; and (V) mean net days of temporary parental leave. A ratio of 1 represents total equality between women and men. For the w/m ratio of employees, salary and education we used data from 2007. Parental leave use can vary significantly between years, especially at small workplaces. Therefore register data from a longer exposure period (2003–2007) was used for both variables on parental leave to ensure reliable measures. Parental leave was measured by Statistics Sweden in the same way each year and a mean value for all years was calculated for each workplace. To make a contextual analysis, these indicators were included in a cluster analysis as described under the section on data analysis. The w/m ratios were categorized in a five-item scale to be suitable for cluster analysis. The cut-off points for the indicators were selected in two steps. In the first step a gender-equal group for each indicator was selected. For all indicators the gender equal group includes the ratio 1 (total gender equality). The allowed deviation from ratio 1 in the gender equal group was defined in relation to the distribution of each indicator (see table 1). For example, in the w/m ratio of employees, a ratio between 0.67 and 1.5 was considered as gender equal. Expressed as a proportion this ratio is equivalent to 40 to 60 percent representation of women at the workplace. In the second step the unequal ratios in both directions of inequality were divided in two equal size groups. This resulted in a five-item scale for each indicator that we used in the cluster analysis:

**Table 1:** Descriptives of ratios for the gender equality indicators with cut-off points for the gender equal group and percentage of workplaces in each gender equality group.

Gender equality indicators	Range	Median	Cut off for the gender equal groups*		Percentage of workplaces in each gender equality group after the categorization				
	min – max	(Inter quartile range)	Ratiow/m	Proportionw/(w+m)	Gender unequal, men higher score (1)	Moderately gender unequal, men higher score (2)	Gender equal (3)	Moderately gender unequal, women higher score (4)	Gender unequal, women higher score(5)
**w/m employees**	0.01–47.00	0.78 (0.21–2.46)	0.67–1.5	40–60	21	21	23	17	17
**w/m salary**	0.00–46.90	0.98 (0.89–1.15)	0.82–1.20	45–55	26	26	41	8	-
**w/m education**	0.38–4.00	0.81 (0.71–0.91)	0.96–1.04	49–51	23	23	20	17	18
**w/m parental leave**	0.01–249.47	2.49 (1.40–3.99)	0.67–1.5	40–60	9	9	14	34	34
**w/m temp. parental leave**	0.06–21.92	1.31 (0.96–.51)	0.82–.20	45–55	12	13	26	25	24

* The gender equal group is centred around a ratio of 1 (equivalent to a 50–50 proportion of women and men).

1. gender unequal ratios with a higher score for men
2. moderately gender unequal ratios with a higher score for men
3. gender equal ratios
4. moderately gender unequal ratios with a higher score for women
5. gender unequal ratios with a higher score for women

### Outcome variable for the cohort

*Psychological distress* (age 42) was chosen as the outcome variable and measured by an index consisting of six items (restlessness, concentration problems, worries/nervousness, palpitations, anxiety and other nervous distress) that the cohort participants had felt during the last 12 months (range 0–6 with higher scores corresponding to more psychological distress). The index was dichotomized (0 = no distress, 1 = one or more items of distress) in accordance with previous research [30] to enable logistic regression analyses. The questions were derived from the Swedish Survey of Living Conditions [31].

### Confounders

Both workplace and individual variables were considered as possible confounders. The variable of *psychological distress* (age 21) among the cohort participants was used as an indicator of health-related selection. Health-related selection implies that earlier psychological distress could affect which workplace a person chooses i.e. that healthy people are selected into gender-equal workplaces. Adjusting for earlier health status is a way to reduce the health-related selection when analysing the health consequence of a gender-unequal workplace [32]. Psychological distress at age 21 was measured with the exact same question and dichotomized in the same way as at age 42.

*Socioeconomic position* (age 42) among the cohort participants was measured with occupation level based on the Swedish SEI classification [33]. Upper white-collar workers (including self-employed) was used as reference category compared to lower white-collar and blue-collar workers.

*Type of work* (age 42) among the cohort participants was measured with three categories of professions based on the Nordic occupational classification: working with people (e.g. health care, education, retail), working with data (e.g. economy, information technology, registration) working with things (e.g. manufacturing, construction, cleaning) [34]. Working with data was used as reference category compared to working with people and working with things.

Register data were collected on age distribution at the workplaces presented as *proportion of employees younger than 38 years old*.

### Data analysis

To identify patterns of gender equality at workplaces (aim 1), hierarchic agglomerative cluster analysis was used on the aggregated workplace data [35]. This method is useful for exploring how different variables coexist and constitute different situations, such as work situations with different risks of ill-health [34]. The hierarchical agglomerative cluster analysis was performed with the SLEIPNER 2.1 software, and Ward's method was used. Data were prepared according to recommendations given by Bergman et al. [36] with imputation of data and multivariate outlier analysis resulting in a final sample of 520 workplaces. The cluster analysis is an iterative process that starts out with all workplaces in separate clusters and ends with all workplaces in one cluster. In each step of the analysis a new cluster solution is produced through merging two clusters (that have the most similar scores on all variables included in the analysis) into one cluster. Similarity is measured by squared Euclidean distance measure (ESS). A low ESS score (>1) indicates a high degree of homogeneity within the cluster, while explained ESS is a measurement of model fitness. If explained ESS is 100 percent, each workplace within each cluster in the cluster solution has identical profiles. In this study a cluster solution with six clusters of workplaces with different patterns of gender equality was chosen and will be presented in the result section. The selection of the cluster solution was based on both statistical and theoretical criteria [36]. First, the distribution of explained ESS was used. Consistent with this, the six-cluster solution fulfilled the criteria of an ESS>50. Second, we also considered the six-cluster solution appropriate as few clusters are preferred when used as predictors. A k-means relocation cluster analysis was performed in order to maximize the explained ESS and homogeneity of the clusters. This was carried out with the Relocate module in SLEIPNER software and resulted in more homogenous clusters and an explained ESS of 52.66.

To analyse how the clusters with different patterns of gender equality were related to psychological distress (aim 2), further analyses were performed on the participants in the cohort. Differences between the clusters in psychological distress were tested by chi-square tests. Multivariate logistic regression analyses were performed separately for women and men to further assess the association between different patterns of gender equality and psychological distress, adjusting for individual socioeconomic position, earlier psychological distress, type of work and age distribution at the workplace. Logistic regression is a robust and often used statistic method in public health research for analysing associations between exposures and unevenly distributed health outcomes. All regression analyses were performed using SPSS Statistics 19 [37] with a significance level at 0.05 and 95 percent confidence intervals.

## Results

**Figure 2:**
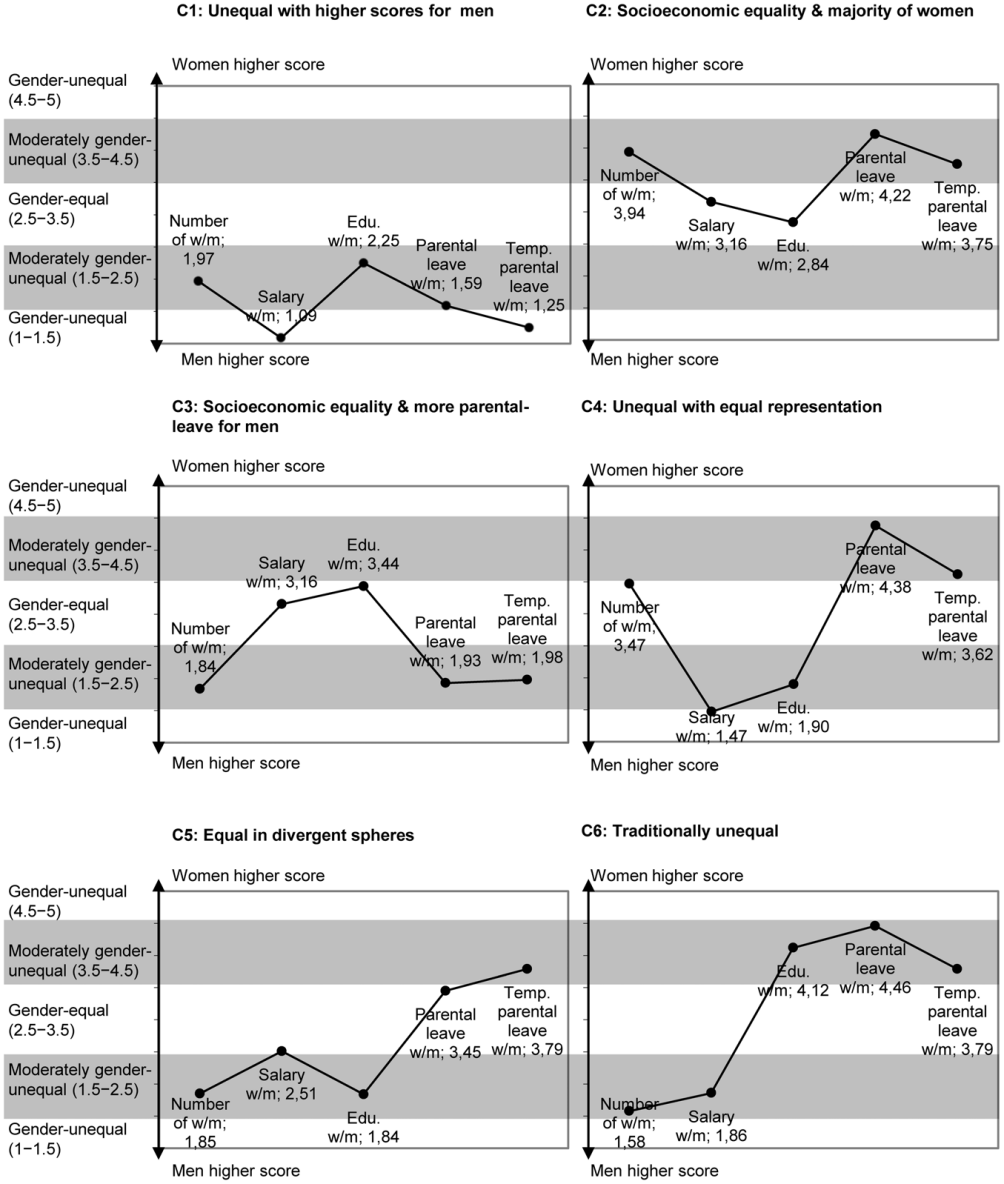
Gender equality patterns for each cluster with mean scores on each gender equality indicator.

The cluster analysis resulted in six clusters of workplaces with different patterns of gender equality. The clusters were named by the main characteristics of their gender equality pattern as presented in Figure 2. No clusters were gender-equal on all five indicators. Four of the clusters were gender-equal in one or more of the indicators (C2, C3, C4, C5) whereas two clusters were gender-unequal on all indicators (C1, C6). The results are structured according to the two aims of the study and presented below.

### Patterns of gender equality at the workplaces (aim 1)

The patterns of gender equality in each cluster are described below in relation to the indicators (Figure 2). The total number of employees from the register data in each cluster and proportion for young employees are described in table 2. For a richer understanding of each clusters, the participants from the Northern Swedish Cohort employed in the clusters are described in terms of socioeconomic position, type of work and occurrence of psychological distress (Table 3).

**Table 2:** Descriptives of all employees at the workplaces for each cluster (n and percent).

Clusters	C1	C2	C3	C4	C5	C6	Total
Number of workplaces	34	140	72	121	73	80	520
Number of employees at workplaces	763	19 108	4 995	62 714	23 146	23 724	134 450
*women*	142	12 086	996	44 666	6 009	4 847	68 744
*men*	621	7 022	3 999	18 048	17 139	18 877	65 706
% young employees (<38)							
*women*	28	30	36	30	33	42*	30*
*men*	37	31	50	31	32	33*	33*

C1: Unequal with higher scores for men.

C2: Socioeconomic equality & majority of women.

C3: Socioeconomic equality & more parental leave for men.

C4: Unequal with equal representation.

C5: Equal in divergent spheres.

C6: Traditionally unequal.

* Significant differences between women and men within the clusters (tested by chi-square test) p<0.05.

**Table 3:** Percentage of cohort participants in each cluster reporting psychological distress and work characteristics (w = women, m = men).

Clusters	C1		C2		C3		C4		C5		C6		Total		p-values: Differences between clusters	
	*w*	*m*	*w*	*m*	*w*	*m*	*w*	*m*	*w*	*m*	*w*	*m*	*w*	*m*	*w*	*m*
N of participants	5	31	132	42	22	59	112	54	32	71	35	120	338	377		
Psychological distress age 21	20	27	28	27	36	34	29	26	23	18	37*	16*	29*	23*	0.77	0.11
Psychological distress age 42	40	32	44	36	24	33	35	29	25	25	51*	20*	39*	27*	0.10	0.29
Change psychological distress (pp)	20	5	16†	9	−12	−1†	6	3	2	7†	24	4	10†	4†		
Socioeconomic position															0.58	<0.001
*Upper white-collar*	20	39	51	64	55*	40*	59	78	66	66	54*	38*	55*	52*		
*Lower white-collar*	40	16	20	24	27*	11*	20	9	12	10	26*	9*	20*	12*		
*Blue-collar*	40	45	29	12	18*	49*	21	13	22	24	20*	53*	25*	36*		
Type of work															<0.001	<0.001
*Working with people*	20	26	62	50	62*	28*	57	37	28	25	9*	13*	51*	26*		
*Working with data*	40	26	33	41	29*	32*	34	50	53	51	60*	34*	38*	39*		
*Working with things*	40	48	5	9	9*	40*	9	13	19	24	31*	33*	11*	35*		
Occupational sector															<0.001	<0.001
*Science & artistic work*	0	10	29*	36*	23	17	10*	15*	19	16	20*	24*	20*	20*		
*Health care*	0	7	28*	10*	18	2	39*	9*	3	1	6*	1*	26*	4*		
*Administration*	40	13	23*	33*	18	12	30*	39*	31	27	34*	10*	27*	21*		
*Commercial work*	20	23	10*	9*	23	19	8*	13*	9	10	6*	9*	10*	13*		
*Transport & communication*	0	3	2*	0*	5	7	2*	5*	13	17	14*	3*	4*	6*		
*Manufacturing*	20	36	0*	5*	5	34	3*	6*	9	11	11*	47*	4*	27*		
*Service*	20	7	8*	7*	5	5	7*	13*	16	18	6*	3*	8*	8*		
*Other*	0	1	0*	0*	3	4	1*	0*	0	0	3*	3*	1*	1*		

C1: Unequal with higher scores for men.

C2: Socioeconomic equality & majority of women.

C3: Socioeconomic equality & more parental leave for men.

C4: Unequal with equal representation.

C5: Equal in divergent spheres.

C6: Traditionally unequal.

† Differences between age 21 and age 42within the clusters (tested by chi-square test) p<0.05.

* Differences between women and men within the clusters (tested by chi-square test) p<0.05.

The workplaces in C1, *Unequal with higher scores for men*, were characterized by a gender equality pattern where men used more days of parental leave and temporary parental leave than women. These workplaces had a majority of men as employees and the men had higher salaries and a higher educational level than women. This cluster had a larger proportion of young men compared to young women. Among participants in the cohort, the most common occupational sector was manufacturing for men and administration for women. For both women and men, the largest group of participants was blue-collar workers and those working with things. Forty percent of the women and 32 percent of the men reported psychological distress at age 42, which was equivalent to the proportions in the total population. Differences between women and men in this cluster were not significant which can probably be explained by the low number of women in this cluster (n = 5).

Cluster 2, *Socioeconomic equality & majority of women*, was the cluster with the highest number of workplaces and therefore represents the most common gender equality pattern in the material. The characteristic feature of the pattern was that women were in the majority and used more days of both types of parental leave than the men at the same workplace, while the salaries and educational level were equal. This was also the only pattern with a majority of women. A high proportion of the cohort participants in this cluster were upper white-collar workers and those working with people. In this cluster women more often worked in the health-care sector whereas men more often worked in scientific and artistic work. This cluster included the largest proportion of psychological distress among men and the second largest proportion among women. For women there was also a significant increase in psychological distress (16 percent units) between age 21 and age 42.

The workplaces in C3, *Socioeconomic equality & more parental leave for men*, were characterized by gender equality in salary and educational level, just like C2. However, in this pattern men were in the majority and used more days of both types of parental leave. Among the participants in the cohort, blue-collar workers were the largest groups among men whereas upper white-collar workers were the largest group among women. The majority of women worked with people, whereas men mainly worked with things. Compared to the other clusters, the psychological distress among cohort women in C3 was low.

The workplaces in C4, *Unequal with equal representation*, were characterized by a gender equality pattern with an equal number of women and men whereas all of the other indicators were unequal. Men had higher salaries and educational level and women used most days of both types of parental leave. Among the participants in the cohort, upper white-collar workers were overrepresented in this cluster. A majority of the women worked with people, whereas working with data was the most common type of work among men. Psychological distress was as frequent as in the total population.

The gender equality pattern of the workplaces in C5, *Equal in divergent spheres*, was characterized by equal salary and equal division of parental leave between women and men. However, women took moderately more days of temporary parental leave. Women were in the minority and had a lower educational level than the men. In spite of that we consider this cluster to be the most gender equal cluster as these workplaces were classified as gender equal in the divergent spheres of economy and parental leave. The age distribution was similar among women and men at these workplaces. In this cluster women and men in the cohort were strikingly similar in the distribution of socioeconomic position, type of work, occupational sector and psychological distress. No significant differences between women and men were found. The largest group of participants were upper white-collar workers, the most common type of work was working with data and the most common occupational sector was administration. In this cluster women and men had a lower proportion of psychological distress compared to the other clusters.

The workplaces in C6, *Traditionally unequal*, had a gender unequal pattern with a majority of men, higher salaries for men, lower educational level for men and fewer days of both types of parental leave for men compared to the women at the same workplace. Among the participants in the cohort, working with data was the most common type of work in this cluster. The proportion of men in the manufacturing sector was significantly higher than among women. There were also significant differences between women and men in socioeconomic position as a majority of women were upper white-collar workers whereas a majority of the men were blue-collar workers. The women in this cluster had the highest frequency of psychological distress compared to women in the other clusters. Men on the other hand had the second lowest frequency of psychological distress compared to other men. Differences between women and men in psychological distress within the cluster were also significant.

### Associations to psychological distress (aim 2)

In the Northern Swedish Cohort (n = 715) psychological distress at age 42 was reported by 39 percent of the women and 27 percent of the men. This was a significant increase of 10 percent units for women and 4 percent units for men compared to psychological distress at age 21 (Table 3). Among the participants in the cohort, there were also significant differences in psychological distress between the clusters (data not shown). However, in separate chi-square analysis for women and men, the differences in psychological distress between the clusters were not significant (Table 3). Multivariate logistic regression analyses were performed with psychological distress as outcome. C5 was used as the reference category as this cluster was gender equal on two indicators in divergent spheres. For men, there were no significant associations with psychological distress in bivariate or multivariate logistic regression analyses (data not shown). For women, belonging to C6 was associated with higher odds for psychological distress in all models except those including psychological distress at age 21 (Table 4). C2 was also associated with higher odds for psychological distress among women adjusting for type of work (model 4) and in the full model (model 6).

**Table 4:** ORs and 95% CIs for psychological distress (age 42) in relation to the clusters among women in the cohort.

	Model 1OR (95%CI)		Model 2OR (95%CI)		Model 3OR (95% CI)		Model 4OR (95% CI)		Model 5OR (95%CI)		Model 6OR (95%CI)	
**Clusters: C5** (ref)	1		1		1		1		1		1	
**C1**	2.00	(0.28–14.20)	2.07	(0.29–14.94)	1.99	(0.27–14.54)	1.87	(0.26–13.44)	1.98	(0.28–14.11)	2.02	(0.27–15.19)
**C2**	2.35	(0.98–5.62)	2.37	(0.99–5.68)	2.23	(0.92–5.42)	**2.67**	(1.09–6.53)	2.32	(0.97–5.54)	**2.51**	(1.01–6.26)
**C3**	0.94	(0.26–3.39)	0.97	(0.27–3.52)	0.78	(0.21–2.90)	1.12	(0.30–4.14)	0.89	(0.25–3.24)	0.95	(0.25–3.63)
**C4**	1.60	(0.66–3.90)	1.63	(0.67–3.97)	1.45	(0.59–3.60)	1.73	(0.70–4.27)	1.61	(0.66–3.93)	1.57	(0.61–4.00)
**C6**	**3.18**	(1.13–8.98)	**3.28**	(1.15–9.30)	2.79	(0.97–8.05)	**2.95**	(1.04–8.41)	**3.09**	(1.09–8.75)	2.72	(0.93–7.92)

Model 1: Bivariate.

Model 2: Adjusted for socioeconomic position.

Model 3: Adjusted for psychological distress age 21.

Model 4: Adjusted for type of work.

Model 5: Adjusted for age distribution at the workplace (proportion of employees <38).

Model 6: Adjusted for model 2, 3, 4 and 5.

## Discussion

In this study we have identified various patterns of gender equality at the workplaces that were associated with psychological distress for women but not for men. For women the highest odds for psychological distress were found on traditionally gender unequal workplaces (C6) and fairly gender equal workplaces with a majority of women (C2). The lowest overall occurrence of psychological distress, as well as same occurrence for women and men, was found at the most gender equal workplaces (C5). The results can be summarized in three main findings according to the hypothesis of this study. First, gender inequality patterns at the workplace are of importance for women's mental ill-health. Second, the results support the convergence theory i.e. that similar working and life conditions are related to similar health outcomes for women and men. Third, the overall results indicate that several dimensions of gender equality need to be taken into account to understand its relation to mental health outcomes.

### Women more affected

Our study showed that the patterns of gender equality at workplaces were related to women's but not to men's psychological distress. The lack of association to psychological distress for men can possibly be related to the relatively small number of participants in each cluster which could result in type two errors. Future research with a larger population is required to rule out associations between gender equality patterns at workplaces and psychological distress for men. Keeping that in mind we will now outline three other plausible explanations for women being more affected.

First, the direction of gender equality might be of importance for why women were more affected by gender inequality at the workplace. In gender unequal situations women are more often disadvantaged whereas men have advantages that can be beneficial for health status. This is illustrated in our analysis as women did not have higher salaries in any of the clusters. Also the most gender unequal cluster (C6) represents a disadvantaged situation for women which had no counterpart for men. The pattern in C6 can be interpreted as a gender regime where women's higher educational level was not economically valued, which could be stressful and harmful for women's mental health. It has been suggested that being in a gendered minority at the workplace can have consequences for ill-health through pathways of increased stress [38]. Our study accentuate that the direction of gender inequality at workplaces is crucial to understanding the different health consequences for women and men.

Second, gender relations at work are situated in a gender order in society including home and family life, where the meaning of parental leave can be different for women and men. Even though both women and men have the right to take parental leave in Sweden, previous research has shown that the workplace culture can influence how the days of parental leave are used, especially for men [22], [23]. At the individual level, previous research has shown that gender equality in parental leave is related to lower occurrence of death and sickness [10], [13]. Our results point in the same direction, as C5 with an equal use of parental leave between women and men at the workplace also had the best mental health status. In addition, the relation between paid work and home seems to be especially important for women's mental health at the women's side of the gender segregated labour market. The high occurrence of mental ill-health among women at workplaces with a majority of women (C2) is both supported [39] and contradicted [14] by previous research in a Swedish setting. A possible explanation could be that that women in this cluster (C2) used more days of parental leave, compared to women in C5, and that a traditional division of parental leave implies more extensive family responsibility for women, which another previous Swedish study has shown to be associated with higher risk of ill-health [10].

A third possible explanation to why gender inequality at workplace was only related to women's mental ill-health could be health-related selection [17], [27]. The results of our study indicate that there could be a negative health selection among women in C6 so that women with psychological distress were selected into these workplaces. However adjusting for previous psychological distress left the odds at a similar high level close to significance, indicating that the health-related selection could not fully explain the higher odds in this cluster. Also, there was no evidence of health-related selection for the other cluster with significant higher odds for psychological distress (C2). At these workplaces with a majority of women the higher odds of psychological distress had accentuated throughout working life (between age 21 and 42) indicating that poor mental health could be a consequence of the work situation. Although health-related selection seemed to be of importance in some situations, it did not fully explain the associations between gender inequality patterns and psychological distress.

### Convergence in health outcomes

Women and men in the most gender equal cluster (C5) were similar regarding work characteristics and as well as psychological distress, giving some support for the convergence theory. Previous research from the U.S. has shown that decreasing differences in women's and men's behaviour and life conditions can explain convergence in health outcomes [11]. Our findings are in accordance with a previous pioneer Swedish study which has shown that gender equality at work is related to more similar levels of sickness absence among women and men [26]. However, in contrast to that study, we also found that the most gender equal cluster (C5) was associated with a low occurrence of psychological distress among both women and men. This indicates that gender equality at the workplace does not only relate to better mental health but also to a convergence in mental health patterns between women and men. The convergence hypothesis has in previous Swedish research gained some support regarding couples gender equality in income and occupation in relation to death and sickness, with low risks for traditional women and un-traditional men [10]. Although there may be complementary explanatory models of the associations between gender equality and health status, our results indicate that the convergence theory is a suitable model for understanding part of the relationship between gender equality and health status in a workplace setting.

### Multidimensional view of gender equality at the workplace

The results showed that patterns of gender equality at workplaces did not follow a simple scale from inequality to equality but were instead characterized by different combinations of gender equality and inequality. In previous public health research the single risk factor of gender segregation of workplaces has been shown to be connected to ill-health in a Swedish context [17], [39]. For women in Sweden and the UK, working at a workplace with a majority of men has been associated with worse health status compared to working at workplaces with other gender compositions [16], [27], [39]. In our analysis women at workplaces with a majority of men only had a higher occurrence of psychological distress when the workplace was unequal in other aspects as well (C6). Also, in the only cluster of workplaces with equal representation of women and men (C4) the work situation was unequal in all other indicators of gender equality. This cluster (C4) also had the same occurrence of psychological distress as the overall population. In all, this indicates that gender equality in numbers alone does not necessarily imply that women and men have the same opportunities, working conditions and mental health outcomes. The characteristics of the clusters therefore highlight the importance of utilizing a multidimensional view of gender equality where several dimensions are taken into account.

### On the method

Our study contributes to the contextual understanding of how patterns of gender equality at workplaces are related to psychological distress. By using the method of cluster analysis, we were able to include the direction of the gender inequalities, i.e. if women or men have a higher score on the measured indicator. Furthermore, the cluster analysis enabled us to consider a combination of several different indicators of gender equality that are at play simultaneously at workplaces [5]. Another strength of this study is the high response rate as well as the longitudinal design of the Northern Swedish Cohort that made it possible to adjust for earlier health status and thereby limit the risk of health-related selection. In this study earlier psychological distress has been measured at age 16, 18, 21 or 30. We chose age 21, to adjust for health-related selection, as the two earlier ages could have been connected with adolescent problems. In this cohort age 21 was an age before the participants were established on the labour market, which is suitable for our topic concerning how gender equality aspects in working life associates to health status. Furthermore, the cohort has proven to be comparable to the country as a whole with regard to socio-demographic and socio-economic factors as well as health status and health behaviours [29].

Although this study has several strengths, there are also limitations that need to be discussed. Psychological distress as well as socioeconomic position is only available for the participants in the cohort and not for all employees at the workplaces. Another limitation is the small number of participants in each cluster, which increases the risk of type two errors. This could possibly explain why there were no significant results for men. For future research other important aspects of gender equality such as hierarchal positions, part-time/full-time employment, job grade and sexual harassment need to be analysed. Unfortunately, such variables are not available in the Swedish registers and could therefore not be included in our analysis. Finally, this study is conducted in a Swedish setting characterized by high women labour market participation, extensive parental leave insurances and a public support for gender equality at the workplace. These specific conditions limit the generalisations of the results to other settings.

## Conclusions

Patterns of gender equality at workplaces do not follow a simple scale from inequality to equality. This study showed that patterns of gender equality at the workplace were related to psychological distress among women only. This can be explained by women's and men's different positions in the gender order, meaning that in gender unequal situations at work it is often women that are disadvantaged whereas men have advantages possibly of benefit for their mental health. Finally, this study supports the convergence hypothesis as gender equality at the workplace did not only relate to better mental health for women but also to more similar levels of mental ill-health between women and men. In order to reduce differences in mental health outcomes for women and men, health policies need to consider gender equality at the workplace level as a social determinant of health. Future research can benefit from a multidimensional approach of gender equality when filling the research gaps of the relation between gender equality at workplace and different health outcomes in various settings.
